# Identification of an obesity index for predicting metabolic syndrome by gender: the rural Chinese cohort study

**DOI:** 10.1186/s12902-018-0281-z

**Published:** 2018-08-06

**Authors:** Leilei Liu, Yu Liu, Xizhuo Sun, Zhaoxia Yin, Honghui Li, Kunpeng Deng, Xu Chen, Cheng Cheng, Xinping Luo, Ming Zhang, Linlin Li, Lu Zhang, Bingyuan Wang, Yongcheng Ren, Yang Zhao, Dechen Liu, Junmei Zhou, Chengyi Han, Xuejiao Liu, Dongdong Zhang, Feiyan Liu, Chongjian Wang, Dongsheng Hu

**Affiliations:** 1 Department of Epidemiology and Health Statistics, College of Public Health, Zhengzhou University, Zhengzhou, Henan People’s Republic of China; 2 The Affiliated Luohu Hospital of Shenzhen University Health Sciences Center, Shenzhen, Guangdong People’s Republic of China; 3 Yantian Entry-exit Inspection and Quarantine Bureau, Shenzhen, Guangdong People’s Republic of China; 4 Department of Preventive Medicine, Shenzhen University Health Sciences Center, Shenzhen, Guangdong People’s Republic of China

**Keywords:** Obesity index, Predict, Metabolic syndrome, Cohort study

## Abstract

**Background:**

To compare the accuracy of different obesity indexes, including waist circumference (WC), weight-to-height ratio (WHtR), body mass index (BMI), and lipid accumulation product (LAP), in predicting metabolic syndrome (MetS) and to estimate the optimal cutoffs of these indexes in a rural Chinese adult population.

**Methods:**

This prospective cohort involved 8468 participants who were followed up for 6 years. MetS was defined by the International Diabetes Federation, American Heart Association, and National Heart, Lung, and Blood Institute criteria. The power of the 4 indexes for predicting MetS was estimated by receiver operating characteristic (ROC) curve analysis and optimal cutoffs were determined by the maximum of Youden’s index.

**Results:**

As compared with WHtR, BMI, and LAP, WC had the largest area under the ROC curve (AUC) for predicting MetS after adjusting for age, smoking, drinking, physical activity, and education level. The AUCs (95% CIs) for WC, WHtR, BMI, and LAP for men and women were 0.862 (0.851–0.873) and 0.806 (0.794–0.817), 0.832 (0.820–0.843) and 0.789 (0.777–0.801), 0.824 (0.812–0.835) and 0.790 (0.778–0.802), and 0.798 (0.785–0.810) and 0.771 (0.759–0.784), respectively. The optimal cutoffs of WC for men and women were 83.30 and 76.80 cm. Those of WHtR, BMI, and LAP were approximately 0.51 and 0.50, 23.90 and 23.00 kg/m^2^, and 19.23 and 20.48 cm.mmol/L, respectively.

**Conclusions:**

WC as a preferred index over WHtR, BMI, and LAP for predicting MetS in rural Chinese adults of both genders; the optimal cutoffs for men and women were 83.30 and 76.80 cm.

## Background

Metabolic syndrome (MetS) [1] is a cluster of metabolic abnormalities highly associated with type 2 diabetes mellitus [2], cardiovascular disease [3, 4], and all-cause mortality [5]. It is also a major and escalating public health and clinical challenge worldwide [6]. The increasing prevalence of MetS is observed all over the world and in China [7–10]. Therefore, the early prediction of MetS is essential to prevent potential severe-cardiometabolic consequences caused by MetS.

Obesity seems to be an underlying risk factor in the development of MetS [11, 12]. Waist circumference (WC) is used as a measure of abdominal obesity, and previous studies suggested that WC could be a powerful tool for predicting MetS [13, 14]. However, other obesity indexes such as weight-to-height ratio (WHtR), body mass index (BMI), and lipid accumulation product (LAP) have been found better predictors of MetS than WC [15–17]. As well, controversy remains as to the superiority and the optimal cutoffs of WC, WHtR, BMI, and LAP for predicting MetS [18, 19].

Many previous studies had a cross-sectional design and sample sizes were small, especially studies in China [20–22]. We used data from a large prospective cohort study to compare the power of WC, WHtR, BMI, and LAP to identify an index for predicting MetS in rural Chinese adults and assess the optimal cutoffs of these indexes for predicting MetS for both genders.

## Methods

### Study design and participants

This prospective cohort study was conducted in rural areas around Luoyang City, Henan Province, in the middle of China. A total of 20,194 participants ≥18 years old were recruited by cluster sampling at baseline (July to August of 2007 and July to August of 2008), and 17,265 participants were followed up (July to August of 2013 and July to October of 2014) (response rate 85.5%).

For this study, we excluded participants with known MetS at baseline (*n* = 6390); incomplete data on anthropometric and laboratory measurements at baseline (*n* = 199); WC ≤ 65 cm for men and 58 cm for women (*n* = 147) (based on the calculation formula of LAP [23]); no follow-up examination or death during follow-up (*n* = 2795); and unknown MetS status at follow-up (2195). Finally, 8468 eligible participants (4085 men) without MetS at baseline were included in the present analysis to identify the baseline obesity indexes to predict the presence of MetS at follow-up.

The study was approved by the Ethics Committee of Zhengzhou University and all participants gave their written informed consent to participate before the start of the study.

### Definition of metabolic syndrome

MetS at baseline and follow-up was diagnosed according to International Diabetes Federation (IDF), American Heart Association, and National Heart, Lung, and Blood Institute (AHA/NHLBI) criteria [24]. The criteria for MetS we used was the presence of 3 or more abnormal values among the following variables: WC (90 and 80 cm for men and women), triglycerides (TG) level (approximately 1.69 mmol/L), high-density lipoprotein-cholesterol (HDL-C) level (approximately 1.04 and 1.30 mmol/L for men and women), systolic blood pressure (SBP; 130 mmHg), diastolic blood pressure (DBP; 85 mmHg) and fasting plasma glucose (FPG; approximately 5.56 mmol/L).

### Data collection and laboratory measurement

Demographic and anthropometric data for each participant were collected by trained investigators who used a standard questionnaire. In the present study, smokers were defined as currently smoking and/or having smoked at least 100 cigarettes during the lifetime; the others were considered non-smokers [25]. Drinking was defined as having consumed alcohol 12 or more times in the previous year. Education level was classified as high school or above and low education level. Physical activity level was classified as low, moderate, and high physical activity level by the International Physical Activity Questionnaire scoring protocol [26].

Weight and height were measured twice to the nearest 0.5 kg and 0.1 cm, respectively, with participants wearing light clothing but no shoes, according to a standard protocol [27]. BMI is an index of general obesity that combines weight and height measurements and is calculated as weight in kilograms (kg) divided by height in meters squared (m^2^) [28]. WC was measured twice at the mid-point between the lowest rib and the iliac crest to the nearest 0.1 cm [29], and WHtR was calculated by dividing WC (cm) by height (cm).

Blood pressure was measured by using an electronic sphygmomanometer (HEM-770AFuzzy, Omron, Japan) according to the AHA standardized protocol [30]. SBP and DBP were measured in triplicate and the results were averaged. Overnight fasting blood samples were collected for assessing levels of total cholesterol (TC), TG, HDL-C, and FPG by using an automatic biochemical analyzer (Hitachi 7080, Tokyo) with reagents from Wako Pure Chemical Industries (Osaka, Japan). Low-density lipoprotein-cholesterol (LDL-C) level was calculated by the Freidwald formula [31].

LAP was calculated by WC and TG concentration as *[WC (cm) – 65] × TG (mmol/L)* for men and *[WC (cm) - 58] × TG (mmol/L)* for women, as proposed by Kahn in 2005 [23].

### Statistical analysis

The baseline data for study participants are described with number (percentage) or mean (standard deviation) for categorical or quantitative variables, respectively. Participants were divided into 2 groups by presence or absence of MetS and differences between the 2 groups were examined by t-tests for continuous variables and chi-square test for categorical variables.

Receiver operating characteristic (ROC) curves [18] were plotted to assess the performance of WC, WHtR, BMI, and LAP in MetS prediction by gender. The model was adjusted for age and the fully adjusted model for age, smoking, drinking, physical activity, and education level. The power of MetS prediction was quantified by the area under the ROC curve (AUC) [21, 22] with 95% confidence intervals (CIs), a larger AUC reflecting better predictive accuracy, and *p*-values for BMI, WHtR, and LAP were computed with WC as the reference measurement. The appropriate cutoffs of the indexes were determined by the maximum of Youden’s index (sensitivity + specificity – 1, with the highest sensitivity and specificity combination).

All statistical analyses involved use of MedCalc 10.1.6.0 (MedCalc Software, Ostend, Belgium). The difference was considered statistically significant at *p*-value < 0.05 based on a 2-sided probability.

## Results

The baseline characteristics of the study population by MetS status at follow-up are in Table 1. Compared to those without MetS men with MetS were younger, less of them were drinkers, and they had lower HDL-C levels. As expected they had higher BMI, LAP, SBP, DBP, TG, and FPG (*p*-value < 0.05). Compared to those without MetS women with MetS were older. As expected the anthropometric and biochemical differences were similar to those in men.

**Table 1:** Baseline characteristics of study participants by metabolic syndrome (MetS) status at follow-up

Variables	Total	With MetS	Without MetS	*p*-value
	(*n* = 8468)	(*n* = 1825)	(*n* = 6643)	
Men	4085 (48.24)	509 (12.46)	3576 (87.54)	
Age (years)	52.38 (13.07)	50.13 (12.77)	52.70 (13.08)	< 0.001
Smoking	2866 (70.16)	357 (70.14)	2509 (70.16)	0.991
Drinking	1063 (26.02)	173 (33.99)	890 (24.89)	< 0.001
Education level				0.228
High school or above	617 (15.10)	86 (16.90)	531 (14.85)	
Physical activity				0.079
Low	931 (22.79)	125 (24.56)	806 (22.54)	
Moderate	724 (17.72)	103 (20.24)	621 (17.37)	
High	2430 (59.49)	281 (55.21)	2149 (60.10)	
WC (cm)	79.59 (7.45)	88.33 (6.66)	78.34 (6.68)	< 0.001
WHtR	0.48 (0.05)	0.53 (0.04)	0.48 (0.04)	< 0.001
BMI (kg/m^2^)	22.55 (2.49)	25.17 (2.46)	22.17 (2.26)	< 0.001
LAP (cm.mmol/L)	21.23 (17.02)	37.11 (20.08)	18.97 (15.25)	< 0.001
TC (mmol/L)	4.29 (0.85)	4.43 (0.86)	4.28 (0.85)	< 0.001
TG (mmol/L)	1.37 (0.66)	1.60 (0.75)	1.34 (0.65)	< 0.001
SBP (mmHg)	123.84 (17.88)	127.82 (19.14)	123.28 (17.62)	< 0.001
DBP (mmHg)	76.65 (10.69)	80.12 (11.13)	76.16 (10.54)	< 0.001
FPG (mmol/L)	5.44 (1.24)	5.59 (1.41)	5.42 (1.21)	0.009
HDL-C (mmol/L)	1.14 (0.25)	1.08 (0.22)	1.15 (0.25)	< 0.001
LDL-C (mmol/L)	2.53 (0.72)	2.62 (0.72)	2.52 (0.71)	0.004
Women	4383 (51.76)	1316 (30.03)	3067 (69.97)	
Age (years)	47.92 (12.45)	49.50 (11.23)	47.25 (12.88)	< 0.001
Smoking	13 (0.30)	4 (0.30)	9 (0.29)	0.953
Drinking	31 (0.71)	8 (0.61)	23 (0.75)	0.607
Education level				0.079
High school or above	337 (7.69)	87 (6.61)	250 (8.15)	
Physical activity				0.065
Low	1358 (30.98)	381 (28.95)	977 (31.86)	
Moderate	1035 (23.61)	316 (24.01)	719 (23.44)	
High	1990 (45.40)	619 (47.04)	1371 (44.70)	
WC (cm)	75.72 (7.85)	81.53 (7.86)	73.22 (6.39)	< 0.001
WHtR	0.49 (0.05)	0.53 (0.05)	0.48 (0.04)	< 0.001
BMI (kg/m^2^)	22.75 (2.93)	24.75 (2.98)	21.89 (2.45)	< 0.001
LAP (cm.mmol/L)	23.10 (15.52)	32.73 (16.85)	18.98 (12.88)	< 0.001
TC (mmol/L)	4.40 (0.89)	4.56 (0.90)	4.33 (0.88)	< 0.001
TG (mmol/L)	1.28 (0.62)	1.42 (0.65)	1.22 (0.59)	< 0.001
SBP (mmHg)	120.47 (19.45)	125.18 (19.53)	118.46 (19.06)	< 0.001
DBP (mmHg)	75.73 (10.59)	79.15 (10.90)	74.27 (10.10)	< 0.001
FPG (mmol/L)	5.37 (1.11)	5.48 (1.26)	5.33 (1.04)	< 0.001
HDL-C (mmol/L)	1.27 (0.27)	1.23 (0.26)	1.29 (0.28)	< 0.001
LDL-C (mmol/L)	2.54 (0.74)	2.68 (0.75)	2.49 (0.73)	< 0.001

Abbreviations: *WC* waist circumference, *WHtR* waist-to-height ratio, *BMI* body mass index, *LAP* lipid accumulation product, *TC* total cholesterol, *TG* triglycerides, *SBP* systolic blood pressure, *DBP* diastolic blood pressure, *FPG* fasting plasma glucose, *HDL-C* high-density lipoprotein-cholesterol, *LDL-C* low-density lipoprotein-cholesterol

Data are number (percentage) or mean (standard deviation)

**Fig. 1:**
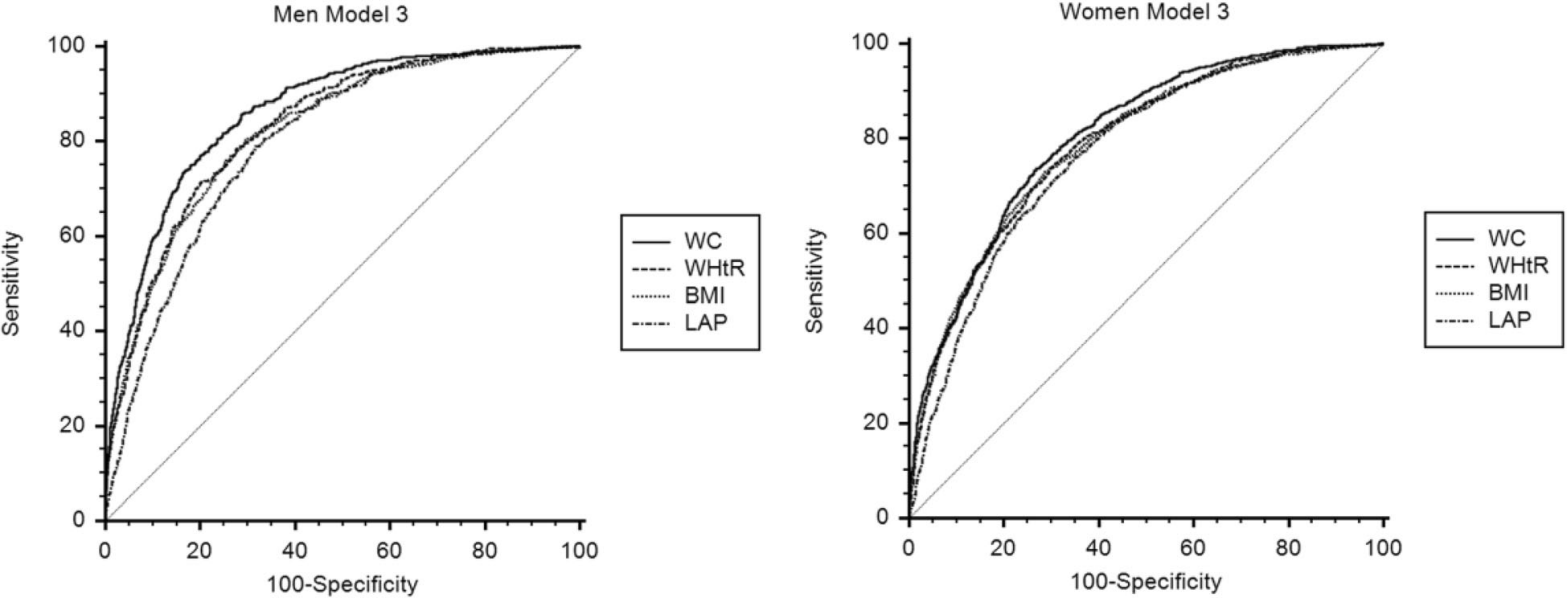
ROC comparing the accuracy of WC, WHtR, BMI, and LAP for MetS for both genders. Abbreviations: ROC, receiver operating characteristic curves; WC, waist circumference; WHtR, waist-to-height ratio; BMI, body mass index; LAP, lipid accumulation product. Model 3: adjusted for age, smoking, drinking, physical activity, and education level

The predictive values for WC, WHtR, BMI, and LAP for MetS for both genders are in Table 2 and Fig. 1. WC, WHtR, BMI, and LAP were all associated with MetS for both genders even after adjusting for age, smoking, drinking, physical activity, and education level. In the unadjusted model (Model 1), WC had the highest AUC value for men and women (0.858, 95% CIs: 0.847–0.868 and 0.804, 95% CIs: 0.792–0.816). On age-adjusted analysis (Model 2), WC was the most accurate for both men and women (0.862, 95% CIs: 0.851–0.873 and 0.805, 95% CIs: 0.793–0.817) and had the highest accuracy in the fully adjusted model (Model 3) (0.862, 95% CIs: 0.851–0.873 and 0.806, 95% CIs: 0.794–0.817). The AUC values for WC, WHtR, BMI, and LAP were all significantly higher for men than women in the unadjusted or adjusted model. According to the results, WC possessed the best power for predicting MetS versus the other 3 indexes on unadjusted and adjusted analyses, with no significant differences between men and women by AUC value.

**Table 2:** AUC values for WC, WHtR, BMI, and LAP for predicting MetS by gender

Model	Variables	Men (*n* = 4085)		Women (*n* = 4383)	
		AUC (95% CIs)	*p*-value⃰	AUC (95% CIs)	*p*-value⃰
Model 1	WC	0.858 (0.847–0.868)	–	0.804 (0.792–0.816)	–
	WHtR	0.819 (0.807–0.831)	< 0.001	0.789 (0.776–0.801)	< 0.001
	BMI	0.821 (0.808–0.832)	< 0.001	0.781 (0.768–0.793)	< 0.001
	LAP	0.796 (0.783–0.808)	< 0.001	0.770 (0.758–0.783)	< 0.001
Model 2	WC	0.862 (0.851–0.873)	–	0.805 (0.793–0.817)	–
	WHtR	0.831 (0.820–0.843)	< 0.001	0.789 (0.776–0.801)	< 0.001
	BMI	0.823 (0.811–0.835)	< 0.001	0.790 (0.778–0.802)	0.012
	LAP	0.798 (0.785–0.810)	< 0.001	0.770 (0.758–0.783)	< 0.001
Model 3	WC	0.862 (0.851–0.873)	–	0.806 (0.794–0.817)	–
	WHtR	0.832 (0.820–0.843)	< 0.001	0.789 (0.777–0.801)	< 0.001
	BMI	0.824 (0.812–0.835)	< 0.001	0.790 (0.778–0.802)	0.010
	LAP	0.798 (0.785–0.810)	< 0.001	0.771 (0.759–0.784)	< 0.001

Abbreviations: *AUC* the area under the ROC curve, *WC* waist circumference, *WHtR* waist-to-height ratio, *BMI* body mass index, *LAP* lipid accumulation product

Model 1: unadjusted model

Model 2: adjusted for age

Model 3: adjusted for age, smoking, drinking, physical activity, and education level

*p-value indicates the statistical significance of other models compared with a model of WC

Table 3 shows the gender-specific optimal cutoffs of WC, WHtR, BMI, and LAP for predicting MetS. The Youden’s index indicated that the appropriate cutoffs of WC for predicting MetS for men and women were 83.30 cm (sensitivity = 81.34%, specificity = 75.62 and Youden’s index = 0.5696) and 76.80 cm (sensitivity = 74.01%, specificity = 72.81% and Youden’s index = 0.4682). The Youden’s index values were highest for WC for predicting MetS for both genders. Additionally, the optimal cutoffs of WHtR, BMI, and LAP for men and women were approximately 0.51 and 0.50, 23.90 and 23.00 kg/m^2^, and 19.23 and 20.48 cm.mmol/L, respectively. The present results suggested that optimal cutoffs were higher for men than women for WC, lower for LAP and similar for WHtR and BMI.

**Table 3:** Optimal cutoffs of WC, WHtR, BMI, and LAP for MetS prediction by gender

Variables	Cutoff	Sensitivity (%)	Specificity (%)	Youden’s index
Men				
WC (cm)	83.30	81.34	75.62	0.5696
WHtR	0.51	73.08	76.43	0.4951
BMI (kg/m^2^)	23.90	72.69	76.99	0.4968
LAP (cm.mmol/L)	19.23	84.28	61.94	0.4622
Women				
WC (cm)	76.80	74.01	72.81	0.4682
WHtR	0.50	74.39	69.61	0.4400
BMI (kg/m^2^)	23.00	71.28	70.33	0.4161
LAP (cm.mmol/L)	20.48	76.44	64.62	0.4106

Abbreviations: *WC* waist circumference, *WHtR* waist-to-height ratio, *BMI* body mass index, *LAP* lipid accumulation product

## Discussion

The present study suggested that WC had the highest accuracy and appropriate cutoffs for predicting MetS for both genders as compared with other obesity indexes such as WHtR, BMI, and LAP even after adjusting for age, smoking, drinking, physical activity, and education level.

In previous studies, the obesity index with the most power for predicting MetS has been widely debated. Among some cross-sectional studies, BMI, WC, and WHtR could similarly predict the presence of multiple metabolic risk factors in Chinese people [32]. However, WHtR was a better index for screening MetS based on the IDF criteria as compared with BMI and LAP for both genders in a Xinjiang population [22]. In a population-based study in China [20], WHtR was the best predictor of MetS in men, but WHtR and WC were equally good predictors of MetS in women. In addition, LAP was a powerful tool for predicting MetS in undiagnosed Brazilian adults [17]. The HANDLS study [14] suggested that WC was the most powerful tool for predicting MetS among adults. Likewise, WC had the highest AUC value as compared with WHtR and LAP in a study of older men and women [33]. Additionally, in a study of adults in northeast China [34], WC was superior to BMI and WHtR in predicting MetS in men, but WHtR was superior to BMI and WC in predicting MetS in women. Among some cohort designs, the San Antonio Heart Study suggested that BMI and WC had equal power in predicting MetS in non-Hispanic whites and Mexican Americans [11]. In contrast, in an Iranian population in the north of Iran, LAP had strong and reliable diagnostic accuracy in predicting MetS, with better predictability than WC, WHtR, and BMI [35]. The Korean Genome and Epidemiology Study [36] suggested that WHtR was a better discriminator of MetS than WC and BMI. The most appropriate index and the accuracy for predicting MetS may depend on ethnicity, age, gender or the diagnostic criteria of MetS, given these inconsistent results.

The present study suggested that the cutoffs of WC for predicting MetS in rural Chinese men and women were 83.30 and 76.80 cm. A population-based study of Chinese people suggested that the optimal cutoffs of WC for men and women were 84.8 and 75.8 cm [21], which is similar to our findings, but lower than the IDF-suggested cutoffs for Chinese men and women of 90 and 80 cm [37]. As well, 2 population-based surveys conducted in China [32, 38] suggested higher WC cutoffs than those in our study. In addition, data from a cross-sectional study of 203 older Brazilians showed WC cutoffs of 90.90 cm for men and 80.20 cm for women [33]. The different ethnicities and race, cross-sectional design, and small sample size may lead to inconsistent results. Further study is needed to explore the application of current WC cutoffs to healthy people in the real world. WHtR cutoffs were 0.51 and 0.53 for Chinese men and women, respectively [32], and our results were the same as these findings and from other studies [34, 38]. The appropriate cutoffs of BMI we found among rural Chinese men and women were approximately 23.90 and 23.00 kg/m^2^, which agreed with findings from population-based studies of Chinese adults [21, 32]. However, the cutoffs of LAP were lower than in previous research. The optimal cutoffs of LAP were previously found to be 34.7 and 27.3 cm.mmol/L for Chinese men and women, respectively [19], and 24.76 and 26.49 cm.mmol/L in the Kazakh adult population in Xinjiang [22]. Additionally, the HANDLS study [14] suggested that optimal cutoffs of various indexes may differ by gender, and we found this trend for WC and LAP but not WHtR and BMI.

The determinant (WC or WC plus TG) is a component of MetS, but in terms of considering the determinant as a potential confounder, we could not consider MetS as a cluster of metabolic abnormalities highly associated with type 2 diabetes mellitus and cardiovascular disease, which occurs together half the time than accidentally alone [24]. As well, although one study showed that confounding occurs in evaluating classification accuracy when a variable is associated with both the marker and the binary outcome [39], many previous studies showed that only age and gender were potential confounders of the association of WC or LAP with MetS [17, 23, 35, 40–42]. We conducted the gender-specific study and adjusted for age in the present study, so the results do not have a bias of accuracy.

The primary purpose of this study was to define the baseline characteristics predicting the presence of MetS at follow-up based on a rural Chinese population. The sample size of this study was not determined specifically; 20,194 cohort members were recruited at baseline examination and followed up for 6 years currently. Therefore, the sample size should be large enough to meet most of the study hypotheses for MetS conditions.

Many previous studies had limitations that included a cross-sectional design [32–34], but our study’s strength lay in its prospective design. Furthermore, we used data from a large population-based-cohort of both genders as compared with previous studies with a small sample size [11, 19–21]. Additionally, we compared various indexes for predicting MetS among rural Chinese adults and provided corresponding AUC and cutoffs for these indexes in this analysis stratified by gender.

We are aware of several relevant limitations of our study besides the strengths we have mentioned. First, although we adjusted for age, smoking, drinking, physical activity, and education level, other confounders might have affected MetS, such as family history of disease, which were not included in the adjustment model. Second, participants were exclusively from rural areas in China, so our results may not be transferred to urban populations. Finally, 2929 participants were not followed up in this cohort, which could imply follow-up bias.

In conclusion, we provide longitudinal evidence for the power of WC, WHtR, BMI, and LAP in predicting MetS, and all 4 indexes were significantly associated with MetS for both genders even after adjusting for some known confounding variables. In addition, WC showed superior power for predicting MetS as compared with the other 3 indexes. The use of a simple index such as WC could contribute to the early prediction of MetS in rural Chinese people, as effective intervention to prevent and treat risks related to MetS. In addition, it may provide useful instruction for public health promotion to maintain optimal cutoffs of WC, BMI, WHtR, and LAP.

Nevertheless, from our results and those of previous research, controversy still remains as to which index has better accuracy for predicting MetS in different countries, ethnicities, and genders. Thus, further larger and prospective research is warranted to elucidate the association between the 4 obesity indexes and MetS and to define appropriate cutoffs in the adult Chinese population.

The WC cutoffs of 83.30 cm for men and 76.80 cm for women from our study are quite a lot different from the 90 cm for Asian American men and 80 cm for Asian American women used in the IDF and AHA/NHLBI criteria [24]. In Japan specific WC cutoffs of 85 cm for men and 90 cm for women were based on visceral fat quantitation on CT scan [43, 44]. Thus country and ethnic specific criteria based on good local data are an appropriate approach. If the cutoffs derived from our data are to be used in practice, we may need to use 83 cm or even 85 cm for men and 77 cm or even 75 cm for women for simplicity but these would still be different from those used for Asian American participants.

## Conclusions

In summary, the present prospective cohort study found that WC at cutoffs 83.30 cm for men and 76.80 cm for women was superior to BMI, WHtR, and LAP for predicting MetS in rural Chinese adults. It is crucial for the early prediction and prevention of MetS to indentify an appropriate index and corresponding optimal cutoffs.

## Abbreviations

AUC: the area under the ROC curve
BMI: body mass index
CIs: confidence intervals
DBP: diastolic blood pressure
FPG: fasting plasma glucose
HDL-C: high-density lipoprotein-cholesterol
LAP: lipid accumulation product
LDL-C: low-density lipoprotein-cholesterol
MetS: metabolic syndrome
ROC: receiver operating characteristic
SBP: systolic blood pressure
TC: total cholesterol
TG: triglycerides
WC: waist circumference
WHtR: weight-to-height ratio
